# Transcriptome analysis revealed the characteristics and functions of long non-coding RNAs in the hypothalamus during sexual maturation in goats

**DOI:** 10.3389/fvets.2024.1404681

**Published:** 2024-06-12

**Authors:** Qing Li, Tianle Chao, Yanyan Wang, Rong Xuan, Yanfei Guo, Peipei He, Lu Zhang, Jianmin Wang

**Affiliations:** ^1^Shandong Provincial Key Laboratory of Animal Biotechnology and Disease Control and Prevention, College of Animal Science and Veterinary Medicine, Shandong Agricultural University, Tai’an, China; ^2^Key Laboratory of Efficient Utilization of Non-Grain Feed Resources (Co-Construction by Ministry and Province), Ministry of Agriculture and Rural Affairs, Shandong Agricultural University, Tai’an, China

**Keywords:** goat, long non-coding RNA, hypothalamus, transcriptome, sexual maturation

## Abstract

The hypothalamus is an essential neuroendocrine area in animals that regulates sexual development. Long non-coding RNAs (lncRNAs) are hypothesized to regulate physiological processes related to animal reproduction. However, the regulatory mechanism by which lncRNAs participate in sexual maturity in goats is poorly known, particularly from birth to sexual maturation. In this study, RNAseq analysis was conducted on the hypothalamus of four developmental stages (1day (D1, *n* = 5), 2 months (M2, *n* = 5), 4 months (M4, *n* = 5), and 6 months (M6, *n* = 5)) of Jining grey goats. The results showed that a total of 237 differentially expressed lncRNAs (DELs) were identified in the hypothalamus. Among these, 221 DELs exhibited cis-regulatory effects on 693 target genes, while 24 DELs demonstrated trans-regulatory effects on 63 target genes. The target genes of these DELs are mainly involved in biological processes related to energy metabolism, signal transduction and hormone secretion, such as sphingolipid signaling pathway, adipocytokine signaling pathway, neurotrophic signaling pathway, glutamatergic synapse, P53 signaling pathway and GnRH signaling pathway. In addition, XR_001918477.1, TCONS_00077463, XR_001918760.1, and TCONS_00029048 and their potential target genes may play a crucial role in the process of goat sexual maturation. This study advances our understanding of lncRNA in hypothalamic tissue during sexual maturation in goats and will give a theoretical foundation for improving goat reproductive features.

## 1 Introduction

The sexual maturity of animals strongly influences their reproductive capacity. Animals go through puberty after birth to reach sexual maturity, a process involving complex physiological changes (1). Early-maturing ruminants experience a younger age of first birth, leading to enhanced reproductive longevity and fecundity (2). The hypothalamus, the gonadal axis’ most upstream tissue and organ, secretes GnRH to stimulate the synthesis of pituitary gonadotropins and gonadal steroid hormones, which are vital for an animal’s sexual development (3–5). The hypothalamus receives signals from the periphery and others that act directly or indirectly on GnRH and its associated reproductive neurons, which in turn affect the synthesis, secretion, and morphology of GnRH, ultimately leading to the occurrence of puberty and sexual maturation (6–8).

Long non-coding RNAs (lncRNAs) are a prevalent class of non-coding RNAs found in mammals, typically exceeding 200 nt in length and devoid of protein-coding capabilities (9, 10). It has been demonstrated to play a significant role in diverse biological processes through transcriptional, post-transcriptional, or epigenetic regulation (11). Currently, lncRNAs have been widely reported to be involved in embryonic development (12), muscle development (13), metabolism (14), and reproductive regulation (15). Recent research has demonstrated that lncRNA Meg3 can regulate the expression of GnRH and Kiss-1 in hypothalamic cells, and knockdown of lncRNA Meg3 can delay puberty in female rats (16). Additionally, the lncRNA MSTRG.33887.2 has the potential to influence goat reproduction by regulating target genes involved in hypothalamic folate metabolism and energy metabolism homeostasis (17). Mouse hypothalamic lncRNA AK044061 plays a crucial role in energy balance by mediating NF-kβ. Neurons with high expression of lncRNAs AK044061 in ARC cells lead to energy imbalance and obesity in mice (18).

However, there is a scarcity of research investigating the dynamic expression patterns of lncRNAs throughout the sexual maturation process in female goats, specifically from birth to the completion of sexual maturation. The Jining grey goat, a well-known high-breeding goat breed in China, exhibits non-seasonal estrus, strong fecundity, and precocious puberty. Sexual maturity in these goats is reached at 3 to 4 months of age, with puberty commencing as early as 2 months of age (19). This trait makes it an ideal animal model for investigating goat fecundity. Hence, it is highly significant to investigate the regulatory mechanism of hypothalamic lncRNAs in the sexual maturation process of female goats.

In this study, the lncRNA of hypothalamic tissue of 1-day-old, 2-month-old, 4-month-old, and 6-month-old (D1, M2, M4, and M6; *n* = 5) female Jining grey goats were sequenced. This study aimed to uncover the expression profile characteristics of lncRNAs during goat sexual maturation, identify lncRNAs associated with hypothalamic development and sexual maturation, and elucidate their molecular regulatory mechanisms. Our research will provide a theoretical basis for the genetic improvement of goat reproductive traits.

## 2 Materials and methods

### 2.1 Animals and sample collection

The experimental goats were all from the Jining Grey Goats Breeding Farm (Jiaxiang, Shandong, China). Under the same feeding management conditions, 20 healthy and disease-free female Jining grey goats were selected. The selected goats were divided into four groups according to age: 1 day old (D1, *n* = 5; body weight (BW): 2.08 ± 0.11 kg), 2 months old (M2, *n* = 5; BW: 4.42 ± 0.24 kg), 4 months old (M4, *n* = 5; BW: 7.62 ± 0.50 kg), and 6 months of age (M6, *n* = 5; BW: 8.82 ± 0.53 kg). The body condition of Jining grey goats was similar in each group. The experimental goats were slaughtered on the same day, and after the electric shock, the hypothalamic tissue was quickly slaughtered and collected, and stored at −80°C.

### 2.2 RNA extraction and library construction

Total RNA was extracted from 20 hypothalamic tissues using TRIzol^®^ reagent (Thermo Fisher Scientific, Waltham, MA, United States). Screening of qualified RNA samples for RNA strand-specific library construction. The rRNA was removed from total RNA samples using the Ribo-Zero rRNA Removal Kit (Illumina, Inc., San Diego, United States), and then a sequencing library was generated using the NEBNext Ultra Directional RNA Library Prep Kit for Illumina (NEB E7420) for Illumina to generate sequencing libraries. The enriched RNA was fragmented using a fragmentation buffer to yield small fragments. Then, the fragmented RNA served as a template for reverse transcription with the addition of 6 bp random primers (random hexamers) to synthesize the first cDNA strand. This was followed by the addition of buffer, dNTPs (with dTTP replaced by dUTP), DNA polymerase I, and RNase H to synthesize the second cDNA strand. The synthesized double-stranded cDNA was purified and enriched via PCR. The PCR product was then purified to obtain the final strand-specific library. After reverse transcription and PCR amplification, 150 bp paired-end reads were sequenced using the Illumina Novaseq6000 platform (Illumina, Inc., San Diego, United States).

### 2.3 Reads mapping and transcriptome assembly

To obtain high-quality sequencing data, we utilized Fastp (v0.23.1) to eliminate sequences containing poly-N, low-quality reads, and adapters from the obtained sequencing data. The high-quality reads obtained are used for downstream data analysis. We generated an index of the reference genome by employing HISAT2 (v2.0.5.) Subsequently, we aligned the clean reads with the goat reference genome (GCF_001704415.2_ARS1.2) using HISAT2 (20). Transcript assembly is performed using Stringtie (v1.3.3b), and gene expression levels are calculated (21). Gene expression levels were normalized using fragments per kilobase of exon model per million mapped reads (FPKM).

### 2.4 lncRNA identification

**Figure 1:**
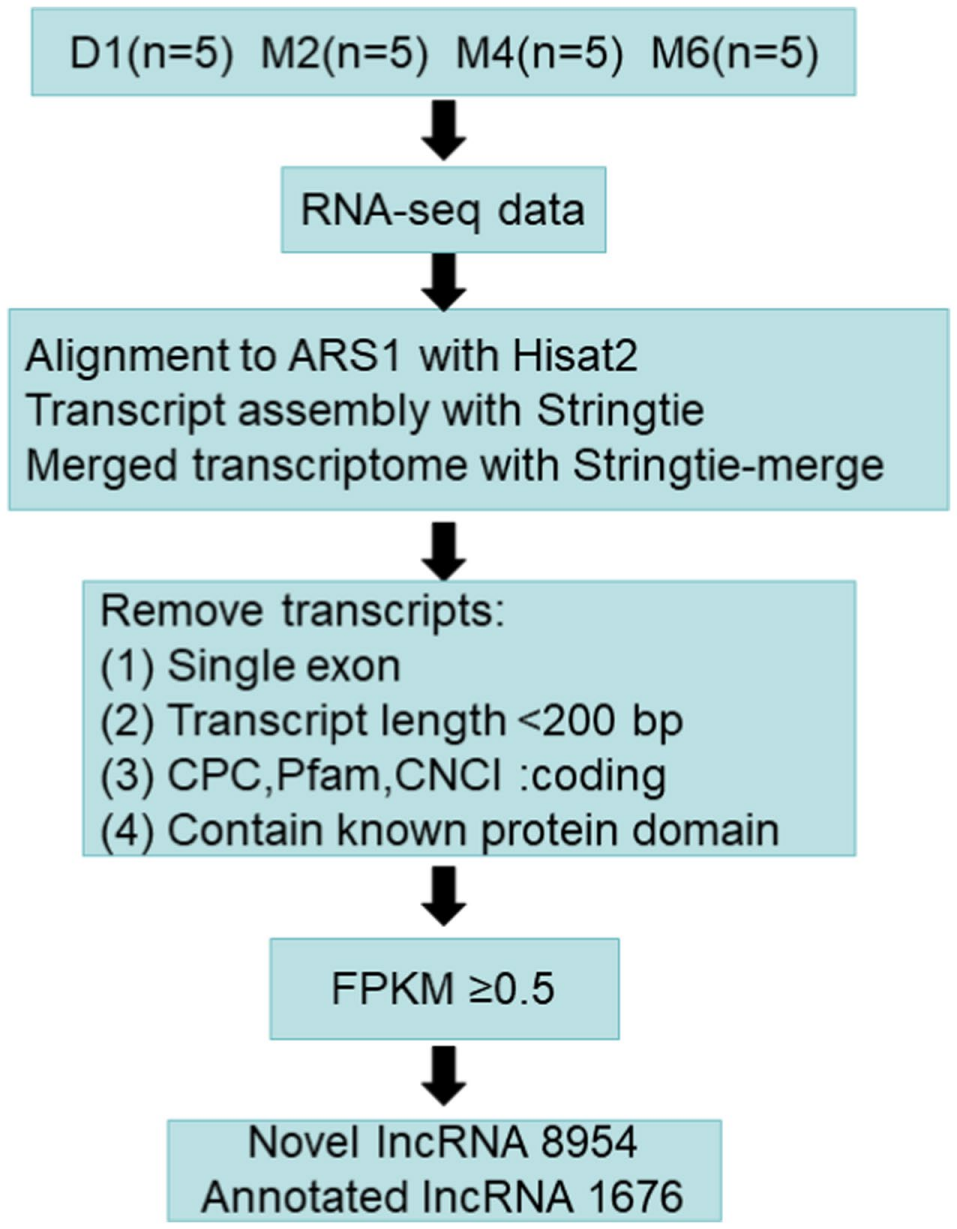
Pipeline for identification of long non-coding RNAs (lncRNAs).

The novel lncRNAs were identified in hypothalamic tissue following the steps shown in Figure 1: (1) removing transcripts with an exon number of 1, (2) removing transcripts less than 200 nt in length, and (3) screening out transcripts that overlapped the exon region annotated in the database by gffcompare software (v0.10.6) (22); (4) CPC2 (v3.2.0) (23), Pfam (v1.6) (24), and CNCI (v2.0) (25) were used to predict the encoding potential of lncRNAs, and the transcripts that were predicted in the three software without coding potential were intersected, and (5) the low-expression lncRNAs (FPKM <0.5) were filtered out.

### 2.5 Differential expression analysis of lncRNAs

The DEseq2 (v1.20.0) package was utilized to examine the differential expression of lncRNAs (DELs) across various developmental stages. The readcounts from the sequencing data were used as the input matrix, and the *p*-value was adjusted utilizing the Benjamini & Hochberg method. The DELs were screened according to the threshold |log_2_ (Fold change)| ≥1 and False Discovery Rate (FDR) <0.05. Cluster analysis of FPKM values of lncRNAs was performed using the ggplot2 package (v3.4.4). Data for lncRNAs were normalized [log_2_(*X* + 1)] and then standardization (*z*-score).

### 2.6 Prediction and functional analysis of potential target genes of lncRNA

lncRNAs can regulate the expression of potential target genes through cis-or trans-regulatory methods. Based on the location information of lncRNAs, mRNAs within 100 kb upstream and downstream of lncRNAs are defined as cis-target genes of lncRNAs (26). There will be a significant correlation with lncRNA expression (|*R*| > 0.95 and *p* < 0.05) is defined as a potential trans-target gene for lncRNA.

Subsequently, we used clusterProfiler software (v3.8.1) to perform Gene Ontology (GO) functional analysis of these differentially lncRNAs predicted target genes (27). The Kyoto Encyclopedia of Genes and Genomes (KEGG) enrichment analysis is performed by KOBAS (http://bioinfo.org/kobas; Accessed: 12.19, 2023) (28). *p* < 0.05 was considered to be significantly enriched.

### 2.7 Quantitative real-time PCR

Six lncRNAs were randomly selected and the accuracy of the lncRNA sequencing results was verified by qRT-PCR. First, we used PrimeScript^™^II First strand cDNA synthesis kit (Takara Bio Inc., Dalian) to reverse transcrib total RNA from goat hypothalamus tissue into cDNA. Then qRT-PCR was performed in a Roche LightCycler 96 using the SYBR PrimeScript^™^ RT-PCR Kit (Takara Bio Inc., Dalian). GAPDH was used as an internal reference gene to correct gene expression levels and normalize the data. The primers designed using Primer 5.0 (Supplementary Table S1). The relative expression levels of lncRNAs were calculated by 2^−ΔΔCT^ (29). One-way ANOVA was performed with SPSS 17.0, and the results were expressed as mean ± standard error. Three repetitions are performed for each set. *p* < 0.05 was considered statistically significant.

## 3 Result

### 3.1 Overview of RNA sequencing data

RNA was isolated from the hypothalamic tissues of female Jining grey goats at four developmental stages (D1, M2, M4, and M6), and 20 lncRNA libraries were constructed. Sequencing of the libraries was conducted on the Noveseq 6000 platform, resulting in a total of 1,803,310,692 raw reads. Following quality control procedures, we obtained 1,764,056,912 clean reads (Supplementary Table S2). The alignment of these clean reads to the goat reference genome was performed using HiSAT2, achieving alignment rates ranging from 86.05 to 95.99%, with unique mapping reads alignment rates between 75.1 and 91.98% (Supplementary Table S2). Subsequent analysis only considered the uniquely mapped reads.

### 3.2 Identification and characterization of lncRNAs

**Figure 2:**
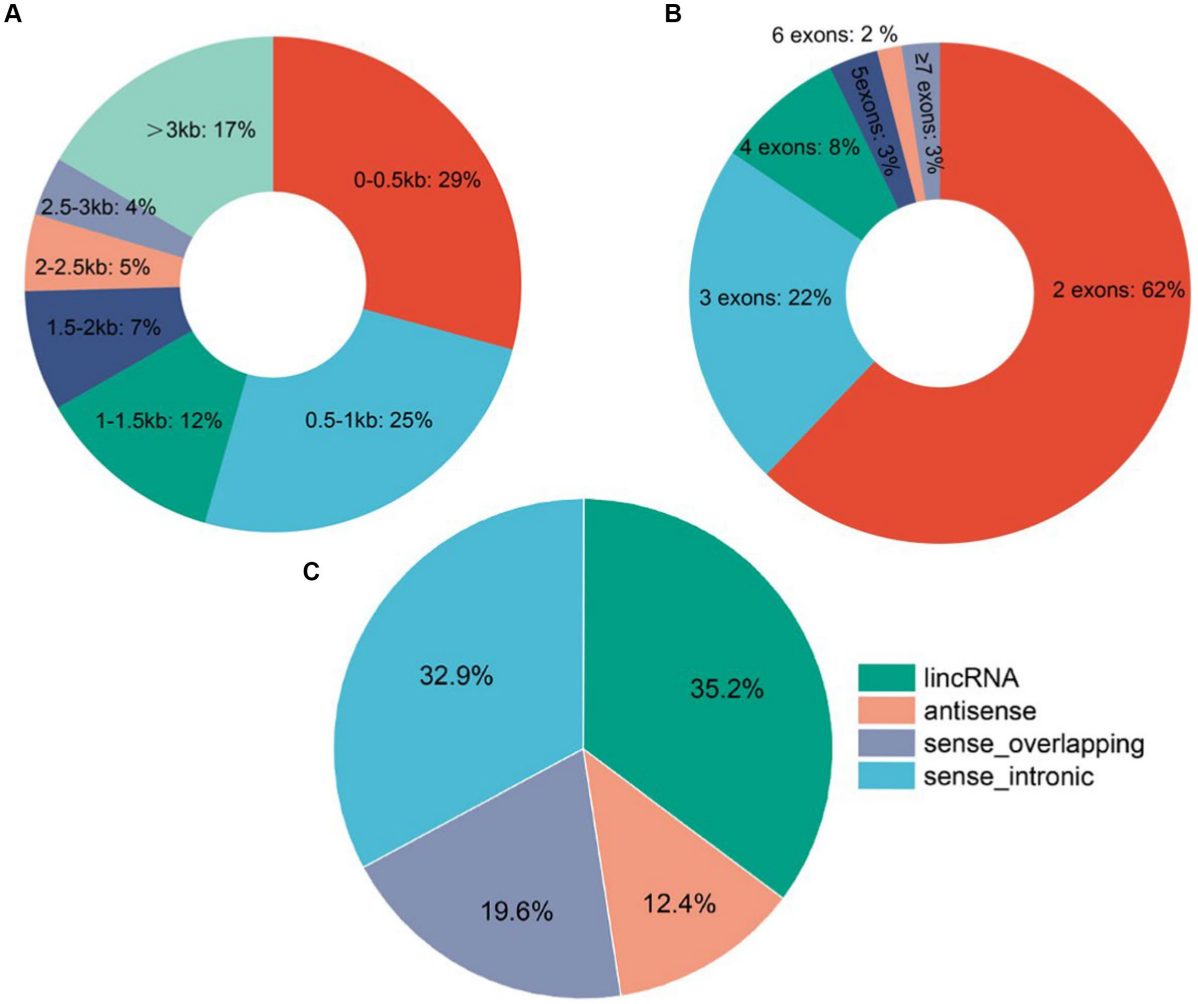
Identification and characterization of goat hypothalamic lncRNAs. **(A)** Pie plot of lncRNA exon number distribution. **(B)** Pie plot of lncRNA length distribution. **(C)** Pie chart of lncRNA classification.

A total of 10,630 lncRNAs were identified according to the steps shown in Figure 1, of which 1,676 were annotated and 8,954 lncRNAs were newly identified. Cluster analysis showed that most of the lncRNAs were expressed at low levels at the D1 stage (Supplementary Figure S1). Further analysis of the identified lncRNA signatures showed that showed that about 62% of the lncRNAs had 2 exons, and a few lncRNAs (3%) had more than 6 exons (Figure 2A). In addition, the length distribution of the identified lncRNAs ranged from 122 to 73,429 bp. More than 50% of the lncRNAs were less than 1,000 bp in length, about 83% were in the 0–3 kb range, and a few (17%) were greater than 3 kb (Figure 2B). About 35.2% of the lncRNAs are located in the intergenic region. Only 12.4% of the lncRNAs are from the antisense region, and about 32.9% of the lncRNAs are from the intron region (Figure 2C).

### 3.3 Differential expression analysis of lncRNAs

**Figure 3:**
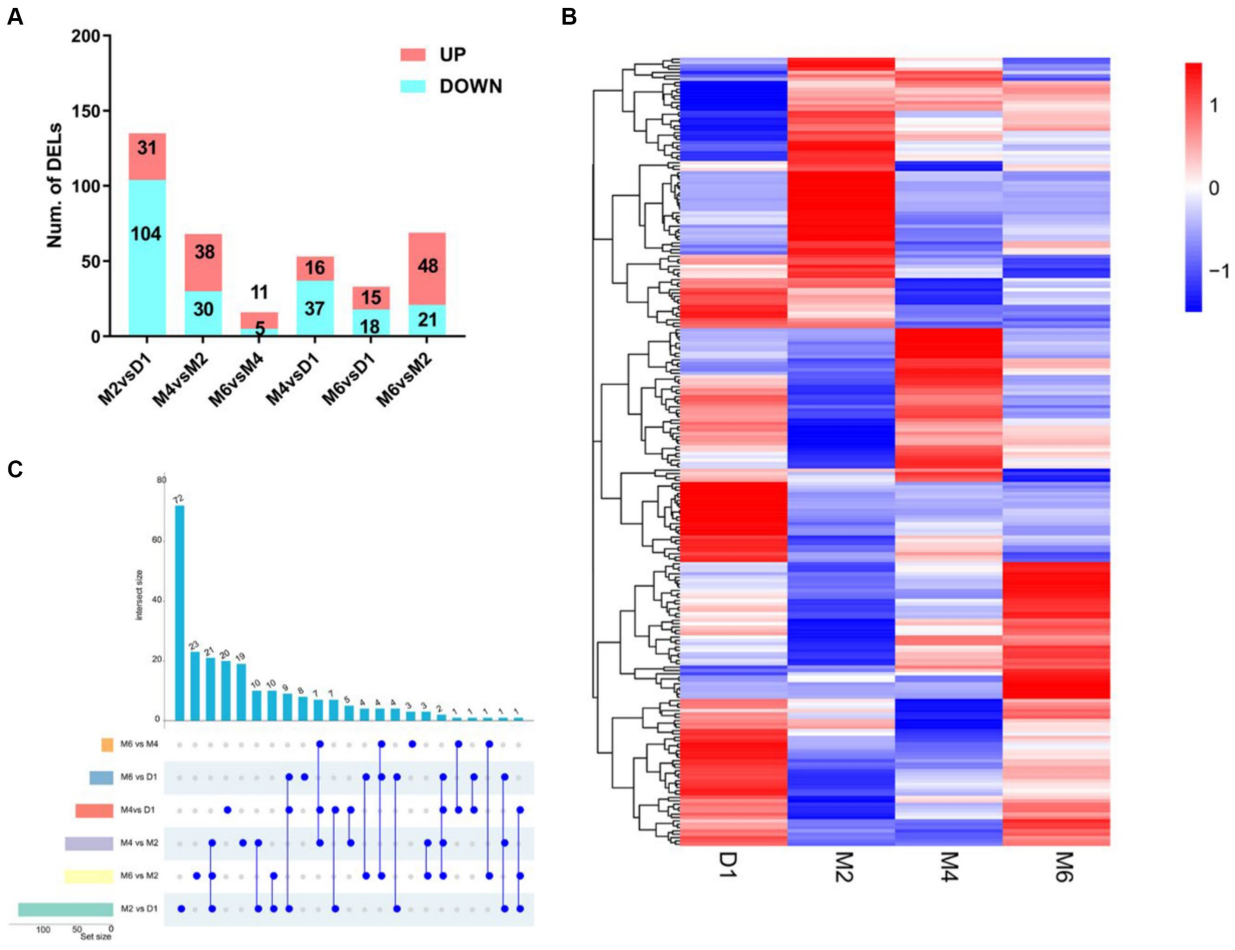
Differentially expresses lncRNA (DELs) characteristics in different comparison groups. **(A)** Cluster analysis of differential lncRNAs. Each column represents a grouping and each row represents a differential lncRNA. **(B)** Histogram of DELs. **(C)** The upset plot shows the distribution of DELs in the different comparison groups.

According to the screening criteria of |log_2_ (Fold change)| ≥1 and *p*_adj_ < 0.05, the lncRNAs of four different developmental stages (D1, M2, M4 and M6) in hypothalamic tissue were compared and analysed. This analysis resulted in the identification of 237 differentially expressed lncRNAs (Figure 3A).

A total of 135 DELs (31 up-regulated and 104 down-regulated) were identified in M2 vs. D1, 68 DELs (38 up-regulated and 30 down-regulated) were identified in M4 vs. M2, and 16 DELs (11 up-regulated and 5 down-regulated) were identified in M6 vs. M4. A total of 53 DELs (16 up-regulated and 37 down-regulated) were identified in M4 vs. D1, 33 DELs (15 up-regulated and 18 down-regulated) were identified in M6 vs. D1, and 69 DELs (48 up-regulated and 21 down-regulated) were identified in M6 vs. M2. Interestingly, Figure 3B shows different expression patterns for these DELs, and Figure 3C shows that the M2 vs. D1 group has the most unique DELs, with 72. The M6 vs. M4 group had the lowest number of DELs with 3. Remarkably, 9 DELs were the same in the three comparison groups of M2 vs. D1, M4 vs. D1, and M6 vs. D1. This suggests that TCONS_00076225, TCONS_00148767, TCONS_00191611, TCONS_00102342, TCONS_00192735, TCONS_00032355, TCONS_00032357, TCONS_00053700, and TCONS_00070238 may be significant in the postnatal sexual development of goats.

### 3.4 lncRNA target gene prediction and functional analysis

lncRNAs have been implicated in influencing gene expression through cis-or trans-interactions. To investigate the role of lncRNAs in the goat hypothalamus, we predicted the potential regulatory roles of the identified DELs on both cis and trans target genes. Specifically, based on a distance threshold of 100 kb between lncRNAs and their target genes, 221 DELs were predicted to regulate 693 target genes in a cis manner (Supplementary Table S3).

**Figure 4:**
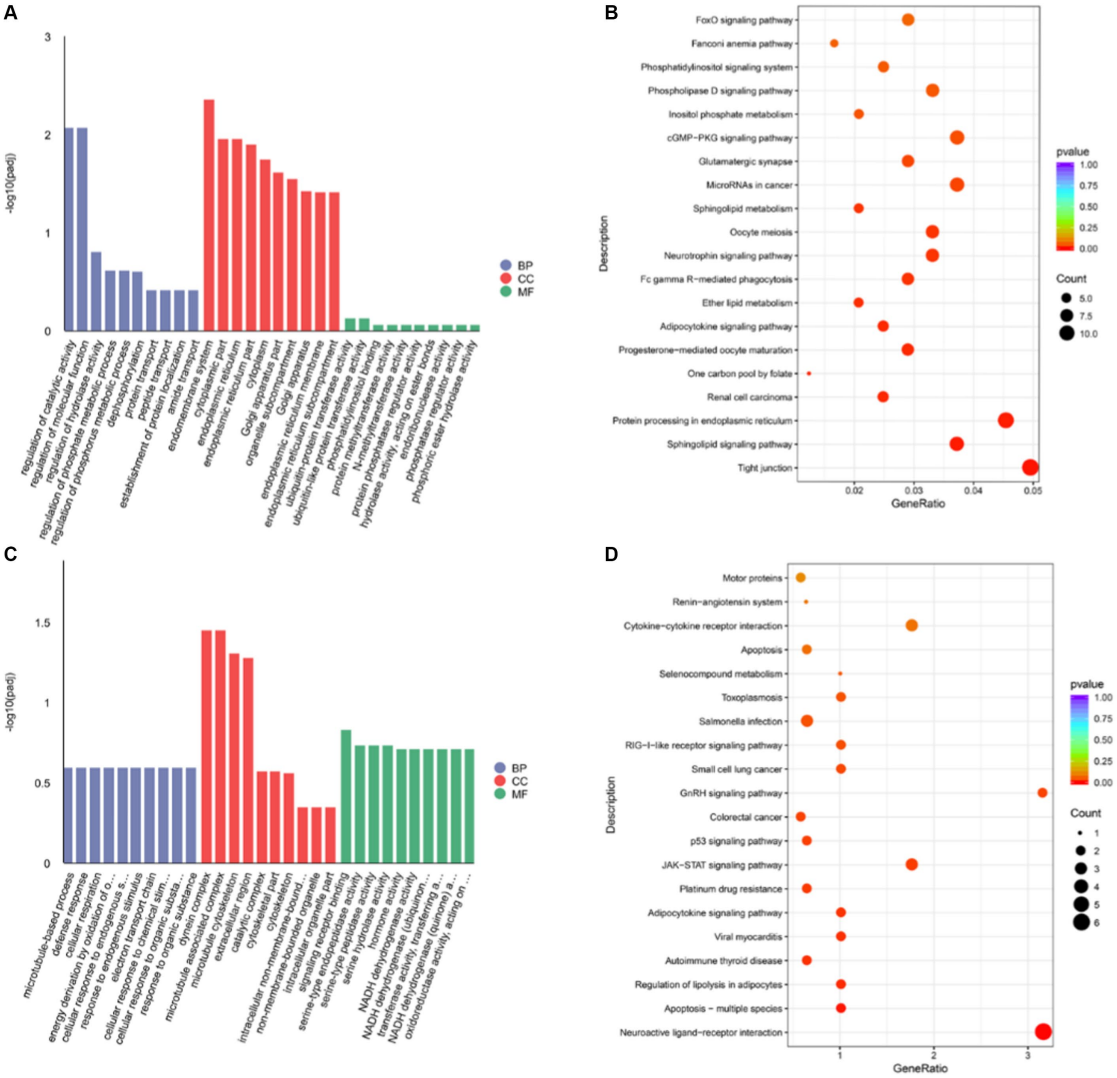
The functional enrichment analysis of DELs target genes **(A)** DELS cis-target gene GO analysis. **(B)** DELS cis-target gene KEGG analysis. **(C)** DELS trans-target gene GO analysis. **(D)** DELS trans-target gene KEGG analysis.

The Gene Ontology (GO) analysis results showed that the target genes were significantly enriched in 50 categories (Supplementary Table S4) These cis-target genes are involved in many biological processes, such as regulation of catalytic activity, regulation of molecular function, regulation of hydrolase activity, and dephosphorylation (Figure 4A). In addition, KEGG analysis showed that these target genes were significantly enriched in sphingolipid signaling pathway, protein processing in endoplasmic reticulum, progesterone-mediated oocyte maturation, adipocytokine signaling pathway, neurotrophin signaling pathway, oocyte meiosis, sphingolipid metabolism, glutamatergic synapse, cGMP-PKG signaling pathway, inositol phosphate metabolism, phospholipase D signaling pathway and other pathways (*p* < 0.05) (Figure 4B and Supplementary Table S5).

According to |*R*| > 0.95 and *p* < 0.05, 24 lncRNAs were found, which had 63 trans target genes (Supplementary Table S6 and Supplementary Figure S2). The number of trans-target genes identified in lncRNAs was significantly lower than that in cis, suggesting that lncRNAs may function mainly by cis-regulating gene expression. The GO analysis results showed that we found that these cis-target genes were involved in many biological processes, such as signal receptor binding, hormone activity, NADH dehydrogenase activity, transferase activity, transfer aminoacyl, etc. (Figure 4C and Supplementary Table S7). In addition, KEGG analysis showed that these target genes were significantly enriched in neuroactive ligand receptors, multiple apoptosis, adipocytokine signaling pathway, GnRH signaling pathway, JAK-STAT signaling pathway, and p53 signaling pathway (*p* < 0.05) (Figure 4D and Supplementary Table S8).

The results suggest that these lncRNAs may be involved in hormone secretion, signal transduction processes, and thus regulation of sexual maturation in goats by modulating cis and trans target genes. Interestingly, LOC108634846, LOC108635405, LOC108637396, AGRP, and PIK3C2G were predicted in both cis and trans target genes. Among these, PIK3C2G is the trans-target gene and the cis-target gene of TCONS_00038560.

### 3.5 Interaction analysis

**Figure 5:**
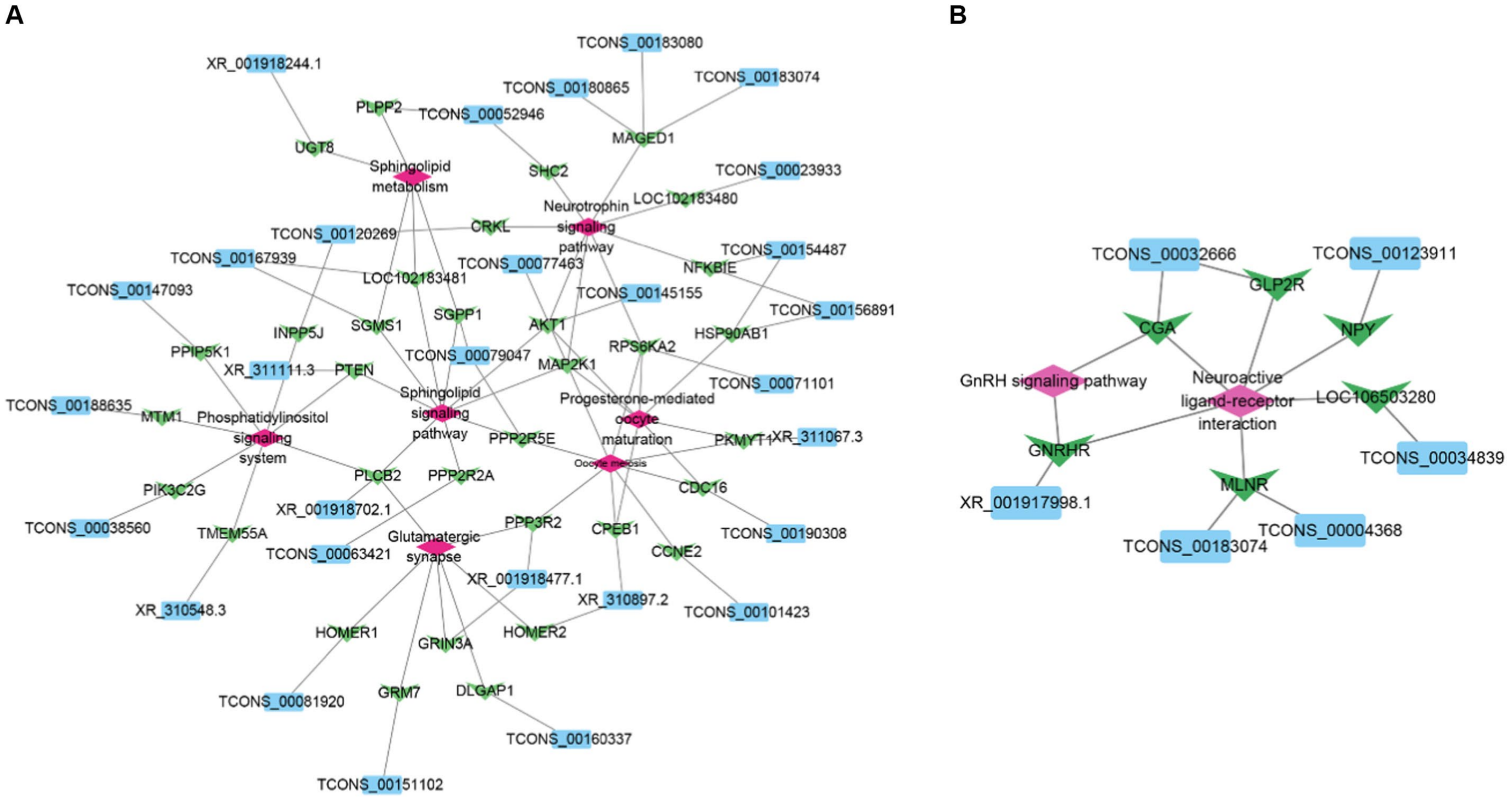
Network diagram of interaction between DELs and target genes. **(A)** Network diagram of interaction between DELs and cis-target genes. **(B)** Network diagram of interaction between DELs and trans target genes. Sky blue represents DELs, light green represents target genes, and pink represents pathway.

To better understand the role of goat hypothalamic DELs in the process of sexual maturation, we selected the target gene of DELs involved in reproduction. Construction of lncRNA-mRNA regulatory networks for DELs and their cis-and trans-target genes, respectively (Figure 5). A total of 31 lncRNAs regulated 34 target genes.

### 3.6 Verify lncRNA expression using qRT-PCR

**Figure 6:**
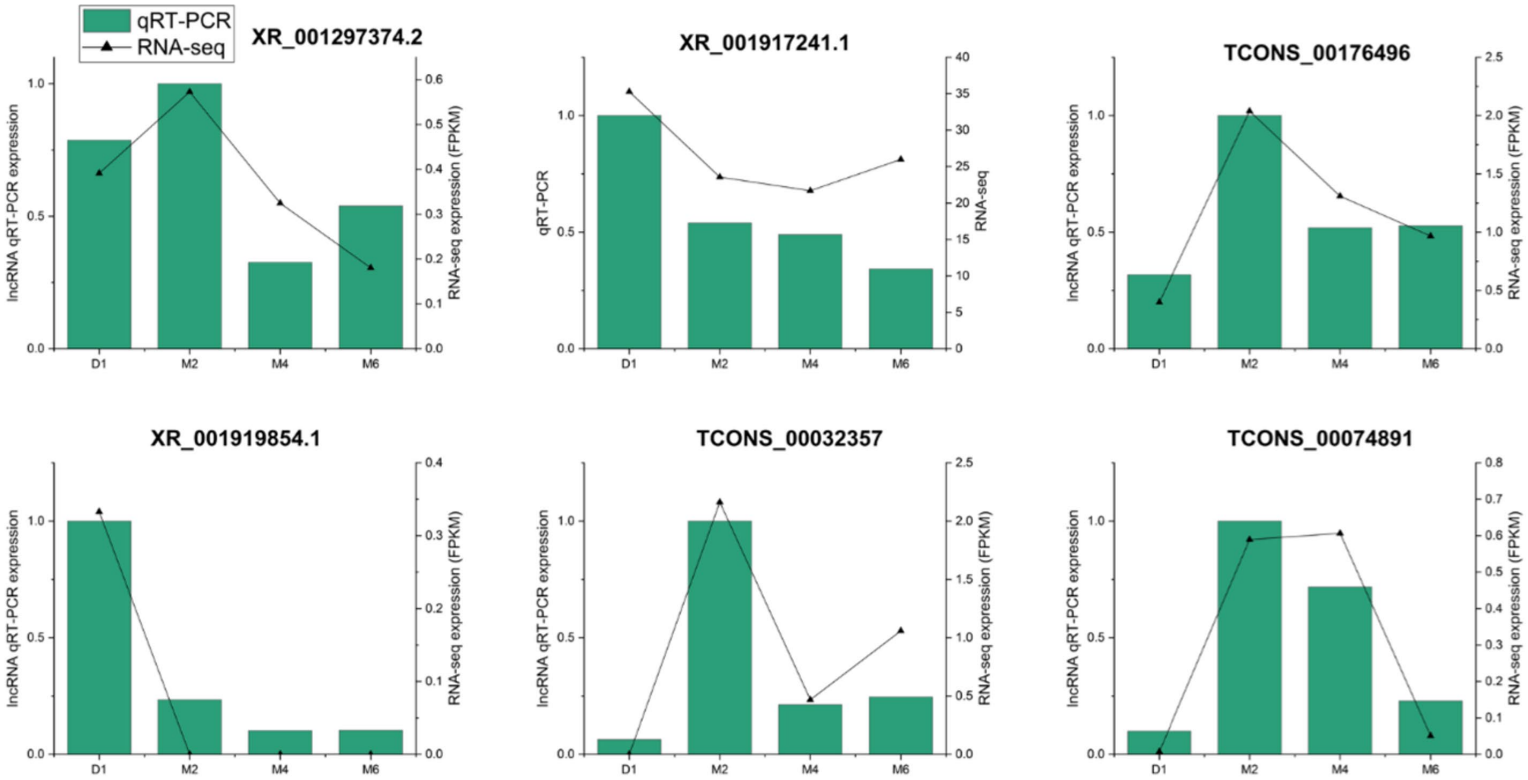
qRT-PCR verification of lncRNA expression levels in hypothalamus at four developmental stages of Jining grey goat.

To verify the accuracy of the sequencing results, we randomly selected 6 lncRNAs (XR_001297374.2, XR_001917241.1, TCONS_00176496, XR_001919854.1, TCONS_00032357, and TCONS_00074891) for qRT-PCR detection. The results showed that the expression patterns of these lncRNAs and those found in the transcriptome data were consistent with the sequencing results (Figure 6). This further illustrates the high reliability and accuracy of RNA-seq.

## 4 Discussion

The hypothalamus is an important neuroendocrine center in mammals and plays an important role in the sexual development of animals. lncRNAs are non-coding RNAs that are more than 200 nt in length and do not have protein-coding functions. Many studies have shown that lncRNAs play an important role in reproductive regulation through transcriptional regulation or epigenetic modification (30, 31). Investigating the role of hypothalamic lncRNAs during sexual maturation in female goats is critical to understanding reproductive mechanisms in this species.

This study conducted lncRNA sequencing on hypothalamic tissue samples obtained from four postnatal developmental stages (D1, M2, M4, and M6) of female Jining grey goats. A total of 10,254 lncRNAs were identified according to rigorous screening criteria, including 1,676 known lncRNAs and 8,954 novel identified lncRNAs. Characterization of lncRNAs revealed that the majority of them consisted of 2–3 exons (84%), and approximately 54% were under 1 kb in length, consistent with previously reported profiles of lncRNAs in goats (32). A total of 236 DELs were identified by differential analysis of lncRNAs. The number of DELs is highest between M2 and D1, totaling 135, while the DELs are least frequent between M6 and M4, with only 16 instances. These results indicated that the number of DELs decreased with the gradual maturity of goats, and the development of Jining grey goats from birth to 2 months of age was an important developmental stage.

Recent studies have shown that lncRNAs are involved in the regulation of gene expression through both cis-and trans-regulatory mechanisms and play an important role in a wide range of biological processes (33, 34). The results showed that 220 DELs regulated 693 target genes through cis. These target genes are involved in the sphingomyelin signaling pathway, progesterone-mediated oocyte maturation, neurotrophin signaling pathway, oocyte meiosis, sphingomyelin metabolism, glutamatergic synapses, cGMP-PKG signaling pathway, phosphoinositide metabolism, and phospholipase D signaling pathway. These pathways are involved in hypothalamic neuronal development, energy metabolism, and reproductive processes (35–38), suggesting that lncRNAs may play an important role in the sexual maturation of goats. Based on this result, we constructed a lncRNA-mRNA regulatory network. Among them, XR_001918477.1, TCONS_00077463, XR_001918760.1, and TCONS_00029048 target GRIN3A, MAP2K1, NOTCH1, and LEP, respectively, and these genes are involved in sexual maturation and reproductive hormone secretion (39–42). GRIN3A is a member of the glutamate-regulated ion channel superfamily, and the expression level of GRIN3A is significantly increased before estrus in mice, which may be related to enhanced glutamate receptor signaling in preovulatory GnRH neurons (43). In mammals, LEP acts on neural circuits in the hypothalamus to regulate feeding and energy metabolism (44), and in addition, leptin can be involved in the regulation of puberty through mTOR (9).

The trans target genes of lncRNAs are predicted by calculating the correlation of mRNA to lncRNAs. This approach enables lncRNAs to regulate mRNA away from their transcription sites (9). In this study, a total of 23 lncRNAs were negatively correlated with 63 protein-coding genes. These target genes are mainly enriched in neuroactive ligand and receptor interactions, JAK-STAT signaling pathway, p53 signaling pathway, and GnRH signaling pathway. Based on this, we speculate that these lncRNAs may be involved in various biological processes, such as hormone secretion and signal transduction, during goat sexual maturation by regulating the expression of these target genes (45–47). Furthermore, we constructed a regulatory network related to reproduction, including 6 lncRNAs and 6 target genes involved in the interaction between neuroactive ligands and receptors and the GnRH signaling pathway. Among them, TCONS_00123911 acts on neuropeptide Y (NPY) through trans regulation. Studies have shown that NPY plays an important role in energy homeostasis and reproductive hormone secretion (48). In addition, NPY has been shown to play an important role in sexual maturation by regulating GnRH secretion patterns and luteinizing hormone secretion (49, 50). In addition, we have identified XR_001917998.1 that can trans-modulate GnRHR. GnRHR plays an important role in the regulation of mammalian reproduction (51). Studies have shown that after hypothalamic GnRH stimulation, GnRHR regulates the activity of the HPG axis through signal transduction, thereby participating in the synthesis and release of LH and FSH, and regulating gonadal function (52, 53).

The identified DELs potentially play a role in the sexual maturation process of goats by modulating target genes through cis-trans regulation. However, experimental validation is required to confirm the functions of lncRNAs. Subsequent research will focus on unraveling the molecular mechanisms through which lncRNAs regulate sexual maturation in goats at both the molecular and cellular levels.

## 5 Conclusion

In summary, we described the characteristics of the expression profile of lncRNA in the hypothalamus at four developmental stages in goats and analyzed the regulatory mechanism of lncRNAs in the process of sexual maturation in goats. In this study, a total of 237 DELs were identified and their cis-trans target genes were predicted, and functional analysis showed that the cis-trans target genes of these DELs were mainly involved in sphingomyelin signaling pathway, glutamatergic synapse, neuroactive ligand and receptor interaction, p53 signaling pathway, GnRH signaling pathway, hypothalamic development and hormone secretion. This work enriches the goat lncRNA database, lays a theoretical foundation for elucidating the molecular mechanism of goat sexual maturation in the future, and will provide a theoretical basis for the improvement of goat genetic traits.

## Data availability statement

The datasets presented in this study can be found in online repositories. The names of the repository/repositories and accession number(s) can be found at: https://www.ncbi.nlm.nih.gov/, GSE244004.

## Ethics statement

The animal study was approved by the Animal Care and Use Committee of Shandong Agricultural University ethics committee (SDAUA-2023-157). The study was conducted in accordance with the local legislation and institutional requirements.

## Author contributions

QL: Conceptualization, Data curation, Formal analysis, Investigation, Methodology, Project administration, Resources, Software, Supervision, Validation, Visualization, Writing – original draft, Writing – review & editing. TC: Funding acquisition, Validation, Visualization, Writing – review & editing. YW: Software, Writing – review & editing. RX: Validation, Visualization, Writing – review & editing. YG: Visualization, Writing – review & editing. PH: Software, Writing – review & editing. LZ: Visualization, Writing – review & editing. JW: Funding acquisition, Investigation, Methodology, Project administration, Writing – review & editing.
