# Supplementary material for: Transcriptome analysis revealed the characteristics and functions of long non-coding RNAs in the hypothalamus during sexual maturation in goats
Source: Front Vet Sci. 2024 Jun 12;11:1404681. doi: 10.3389/fvets.2024.1404681 (PMC11210318; doi:10.3389/fvets.2024.1404681)
Supplement: Supplementary file 1 [file Image_1.pdf]

Supplementary Material

1.1 Supplementary Figures

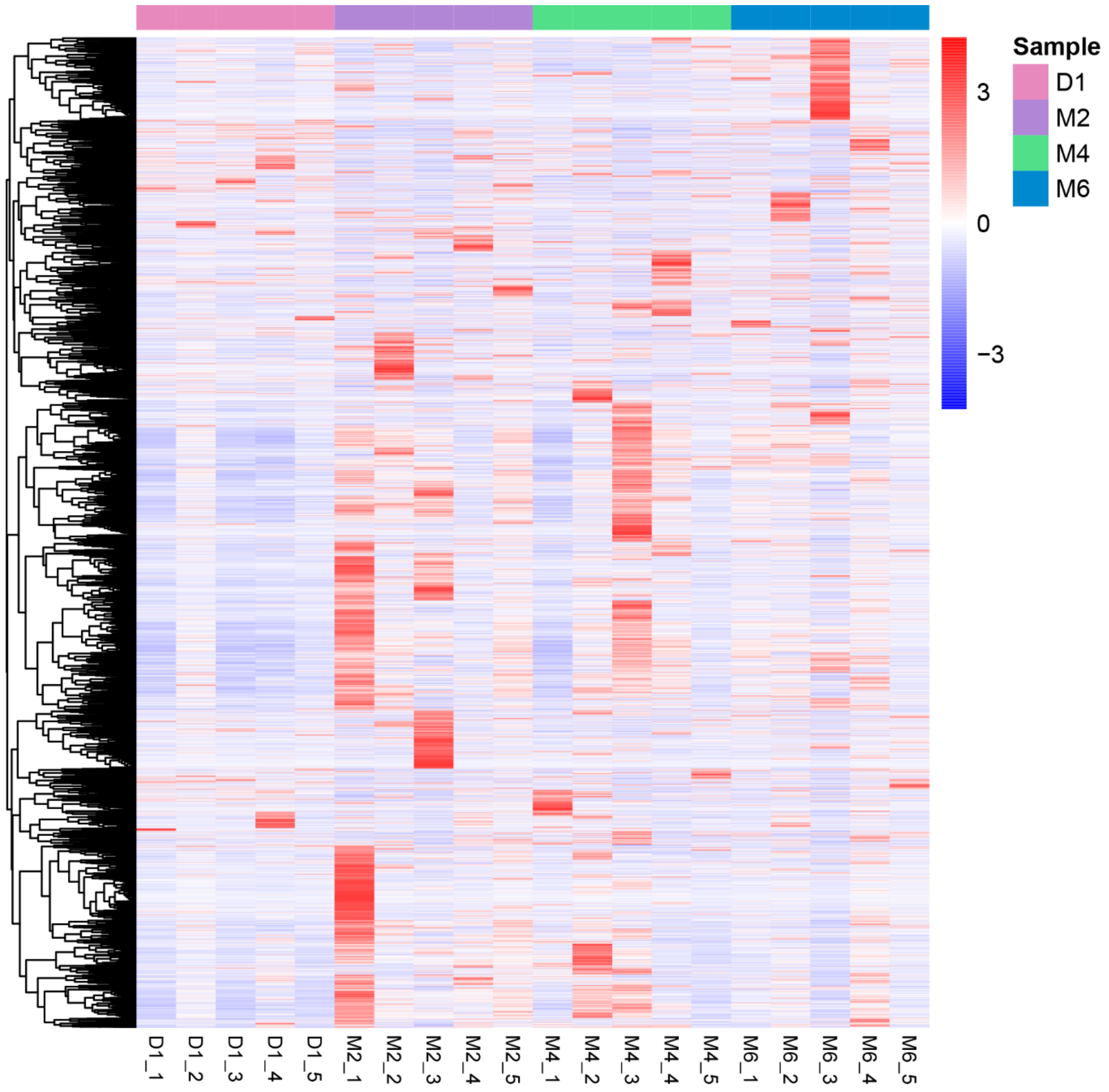

Supplementary Figure 1. Heatmap of lancRNA cluster analysis of hypothalamus at different developmental stages of Jining grey goats. D1, 1 day; M2, 2 month; M4, 4 month; M6, 6 month.

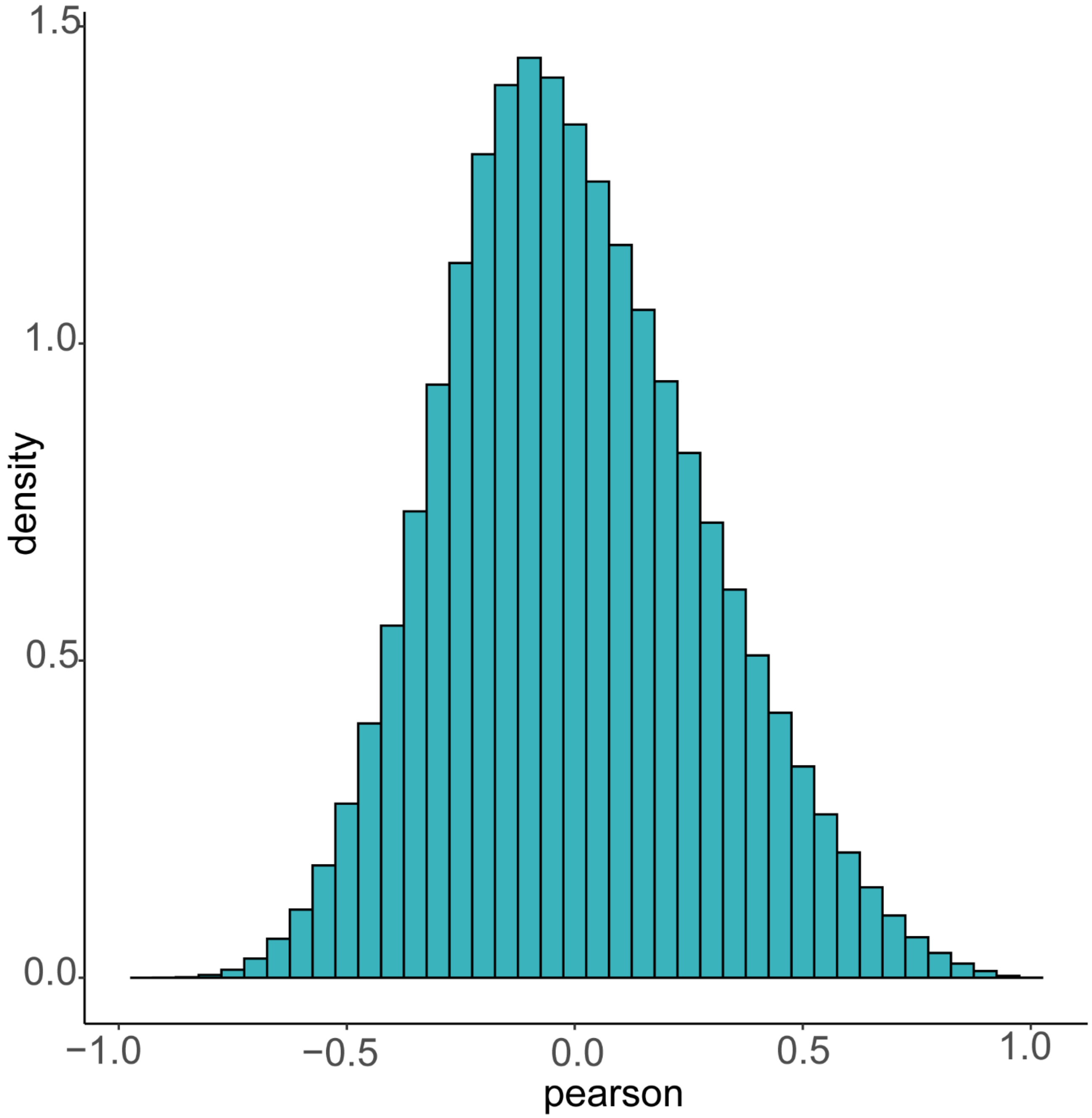

Supplementary Figure 2. Histogram of the correlation of the lncRNA trans target gene prediction.
